# Oyster reef restoration facilitates the recovery of macroinvertebrate abundance, diversity, and composition in estuarine communities

**DOI:** 10.1038/s41598-022-11688-6

**Published:** 2022-05-17

**Authors:** Adam R. Searles, Emily E. Gipson, Linda J. Walters, Geoffrey S. Cook

**Affiliations:** 1 Department of Biology, University of Central Florida, 4000 Central Florida Blvd., Orlando, FL 32816 USA; 2 Institute of Food and Agricultural Sciences, School of Natural Resources and Environment, University of Florida, Gainesville, FL 32603 USA; 3 Skidaway Institute of Oceanography, University of Georgia, 10 Ocean Science Cir, Savannah, GA 31411 USA

**Keywords:** Restoration ecology, Marine biology, Community ecology

## Abstract

Historic declines in oyster populations have resulted in diminished production of ecosystem services and habitat function in many estuaries. Due to the important role of oysters in ecosystem function, scientists and resource managers have employed oyster reef restoration to mitigate declines, recover essential ecosystem services, and better habitat function. Yet, there are knowledge gaps regarding the impact of restoration efforts on ecologically valuable mid-trophic level organisms inhabiting these systems. To address this knowledge gap, here we quantify macroinvertebrate species abundance, community diversity, and composition on experimental restored oyster reefs before and after restoration, and from live (positive control) and dead (negative control) reefs in the Indian River Lagoon, Florida. Species diversity and composition on restored reefs shifted towards states similar to live (positive control) reefs within 12 months of restoration. Recovery of species abundance occurred within 18 months of restoration. The results presented herein quantify the effect of restoration on resident macroinvertebrates and provide timelines of recovery for each attribute of these communities. Further, this study presents an actionable and transferable framework for identifying effective single-species metrics of restoration success across ecosystems. The application of this framework will provide managers and researchers with tools to improve the efficiency and efficacy of post-restoration monitoring. By doing so, this study contributes significantly to the improvement of broader restoration practices in an era of unprecedented habitat loss.

## Introduction

Estuaries are one of the most productive and valuable ecosystems in the world^1–3^. The vast array of habitats and diverse fauna inhabiting these coastal areas produce myriad ecosystem services such as fisheries production, water filtration, recreational opportunities, and coastal storm protection^1,3^. Due to their many benefits and resources derived by human communities, coastal zones have been greatly altered, and estuaries have become one of the most imperiled biological zones^2–4^. Human modification and use of coastal zones has resulted in habitat destruction and degradation via overfishing, eutrophication, and land use development^2,5,6^. These pressures have compounded to threaten the productivity and resilience of estuarine ecosystems and fundamentally alter and degrade significant portions of ecologically and economically valuable habitats^2,5–7^.

Habitat loss is arguably the greatest threat to biodiversity and ecosystem function^8–11^. Up to 50% of terrestrial and marine habitats have been modified or lost globally^1,2,5,11^. Widespread habitat loss, whether a result of direct destruction or indirect degradation and modification, facilitates declines in species abundance and significantly increases the probability of species extinction^8,12^. Species extinctions may adversely affect ecosystem function by potentially removing ecosystem engineers, strong interactors in food webs, and species possessing influential functional traits^9^. Further habitat loss may accelerate declines in ecosystem function as species functional redundancy is reduced over time^9,13^. Pervasive habitat loss and degradation thus threaten ecosystem productivity and the production of essential ecosystem services, posing significant risk to human populations^9^. Thus, scientists and natural resource managers have invested much effort in the field of restoration ecology, with the goal of shifting degraded habitats and ecosystems to an improved state^14^. By altering the physical environment in ways that benefit local foundation species, restoration may effectively facilitate the recovery of local biodiversity and ecosystem function^15,16^.

Oysters are a foundation species and among the most valuable contributors to estuarine function and productivity. As oyster reefs grow, they create complex three-dimensional structure that provides high-quality habitat for many organisms^17–19^. These intricate habitats support unique communities of benthic organisms compared to other estuarine habitats including seagrass beds and bare-bottom sediments^20–22^. Due to these unique communities, oyster reefs also support complex food webs, generating the greatest proportion of secondary production of all estuarine habitats^23^. Reef-associated invertebrates efficiently transfer energy from basal resources to higher trophic level predators, thus contributing significantly to fisheries production and broader ecosystem function^23–26^. However, 85% of oyster reefs across the globe have disappeared entirely, severely crippling the ability of these communities to support estuarine food webs^27,28^. Immense efforts have been undertaken to restore oyster abundances across the globe with the goal of recovering vital ecological functions^28^. Understanding the efficacy of these habitat restoration efforts in facilitating the recovery of the unique ecological communities inhabiting oyster reefs, is vital to achieving the goal of broader estuarine function.

The importance of reef resident invertebrates to higher trophic levels necessitates investigation into the effects of oyster reef restoration on these species. Several studies have investigated the community dynamics of oyster reef invertebrates post-restoration^29,30^. However, a thoroughly controlled and replicated experiment aimed at assessing the response of resident invertebrates to the restoration of dead reefs is lacking in the scientific literature. Furthermore, the identification of transferable and reliable metrics of restoration success that can be applied to broader restoration efforts across multiple ecosystems will increase the ability of practitioners to rapidly assess restoration success. To address these needs here we 1) quantify changes in macroinvertebrate abundance, diversity, and community composition post-restoration, 2) determine the temporal lag between restoration and the recovery of each of these community metrics, and 3) identify indicators of restoration success to guide future restoration efforts. The knowledge generated by completing this study and achieving the aforementioned objectives will yield valuable insight into the effects of habitat restoration on functionally influential components of estuarine food webs. Additionally, this information will improve future restoration and monitoring efforts by providing attainable goals for easily measurable community metrics.

## Results

### Species diversity

Live oyster reefs supported a mean of 632.60 ± 50.44 oysters/m^2^ in the intertidal zone, whereas dead reefs supported a mean of 16.0 ± 1.62 oysters/m^2^ and consisted of disarticulated oyster shell mounds elevated well above the mean high tide line. Reefs belonging to these treatments were 261.782 mm and 148.217 mm thick, respectively. The term “restored reefs” refers to dead reefs that were assigned to the restoration treatment and subsequently restored to monitor changes in macroinvertebrate communities post-restoration. Oyster density on reefs restored in 2017 increased from 0 before restoration to 347.40 ± 45.0 oysters/m^2^ 24 months later. Thickness of these reefs increased from 177.6 ± 4.838 mm to 339.45 ± 12.276 mm in the same time period. Reefs restored in 2018 increased from 0 to 84.0 ± 5.60 oysters/m^2^ and from 27.73 ± 2.22 mm to 61.975 ± 2.355 mm thick by the 12-month sampling period. Live and dead reefs served as positive and negative controls, respectively, to isolate and quantify the effects of restoration on these resident populations.

In this study 20,087 marine invertebrates belonging to 41 species across 14 families were collected from all intertidal oyster reefs (Table 1). A total of 5538, 6866, and 7683 individuals were collected from the live, dead, and restored treatments, respectively. *Petrolisthes armatus* (green porcelain crab Gibbes 1850) was the most abundant species across all treatments (n = 12,534), followed by *Eurypanopeus depressus* (n = 2736; flatback mud crab Smith 1869) and *Rhithropanopeus harrisii* (n = 1539; Harris mud crab Gould 1841). Thirty-six species were collected from live reefs while 29 and 27 species were observed on restored and dead reefs, respectively. The impact of these treatments on diversity over time was dichotomous for reef interiors and margins. In general, restoration treatment had a large effect on invertebrate diversity on reef interiors whereas few noticeable trends were observed on reef margins. This pattern was consistent across reefs studied in both 2017 and 2018. Thus, the focus of our study shifted to post-restoration community dynamics on reef interiors.

**Table 1:** Abundance of species by family collected from oyster reefs over the course of the study.

Species by family					
**Alpheidae**		**Menippidae**		**Penaeidae**	
*Alpheus heterochaelis*	413	*Menippe mercenaria*	49	*Farfantepenaeus aztecus*	4
*Alpheus *spp.	22	**Palaemonidae**		*Farfantepenaeus brasiliensis*	1
*Synalpheus *spp.	2	*Leander *spp.	51	*Farfantepenaeus duorarum*	7
**Amphiuridae**		*Macrobranchium *spp.	80	*Farfantepenaeus *spp.	1
*Amphiodia pulchella*	1	*Palaemon *spp.	264	*Litopenaeus setiferus*	1
*Amphipholis squamata*	41	*Periclimenaeus *spp.	185	**Porcellanidae**	
*Amphiura *spp.	1	*Periclimenes *spp.	7	*Petrolisthes armatus*	12,534
**Diogenidae**		**Panopeidae**		*Petrolisthes galathinus*	59
*Clibinarius tricolor*	2	*Eurypanopeus depressus*	2736	**Portunidae**	
*Clibinarius vittatus*	60	*Eurypanopeus dissimilis*	1	*Callinectes ornatus*	46
**Epialtidae**		*Eurytium limosum*	1	*Callinectes sapidus*	42
*Libinia dubia*	16	*Dyspanopeus sayi*	275	*Callinectes similis*	14
**Eunicidae**		*Panopeidae *spp.	1	*Callinectes *spp.	3
*Marphysa sanguinea*	2	*Panopeus herbstii*	1039	*Charybdis hellerii*	14
**Grapsidae**		*Panopeus simpsoni*	357	**Sesarmidae**	
*Pachygrapsus gracilis*	4	*Rhithropanopeus harrisii*	1539	*Armases cinerum*	1
**Hippolytidae**				*Sesarma curacaoense*	3
*Hippolyte *spp.	204				
*Latreutus *spp.	4				

*Petrolisthes armatus* was the most abundant organism observed, with 12,534 individuals collected. The Porcellanid was followed by *Eurypanopeus depressus* (2736) and *Rhithropanopeus harrisii* (1539).

**Figure 1:**
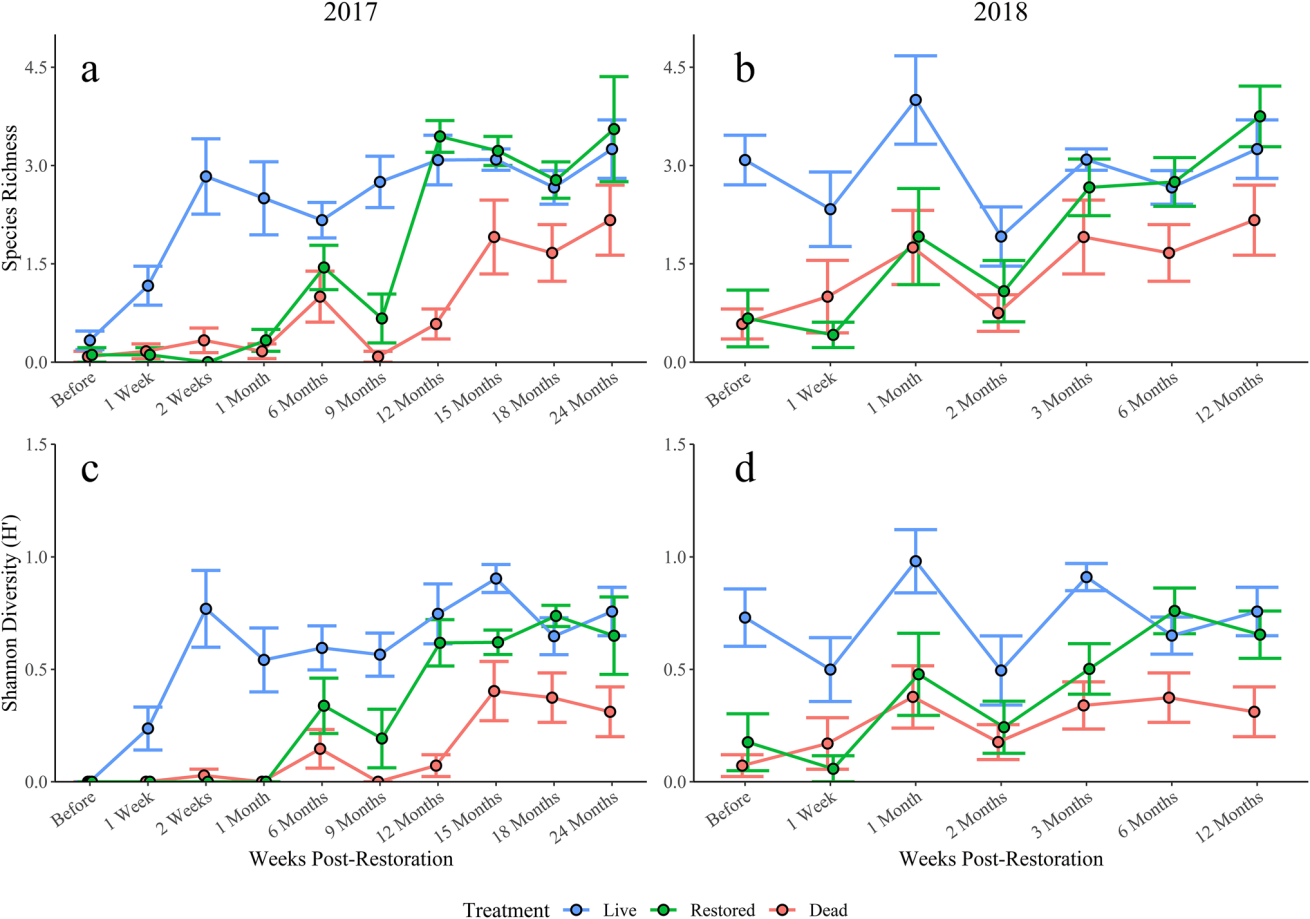
Species richness (top row) and Shannon diversity (bottom row) on 2017 (left) and 2018 (right) reefs. Error is shown for each treatment-time point as ± SE. Species richness and Shannon diversity on restored reefs recovered to states equivalent to those on life reefs as soon as 6 months after restoration. Diversity indices recovered fully by 12 months post-restoration and mirrored live reefs for the remainder of the study.

**Figure 2:**
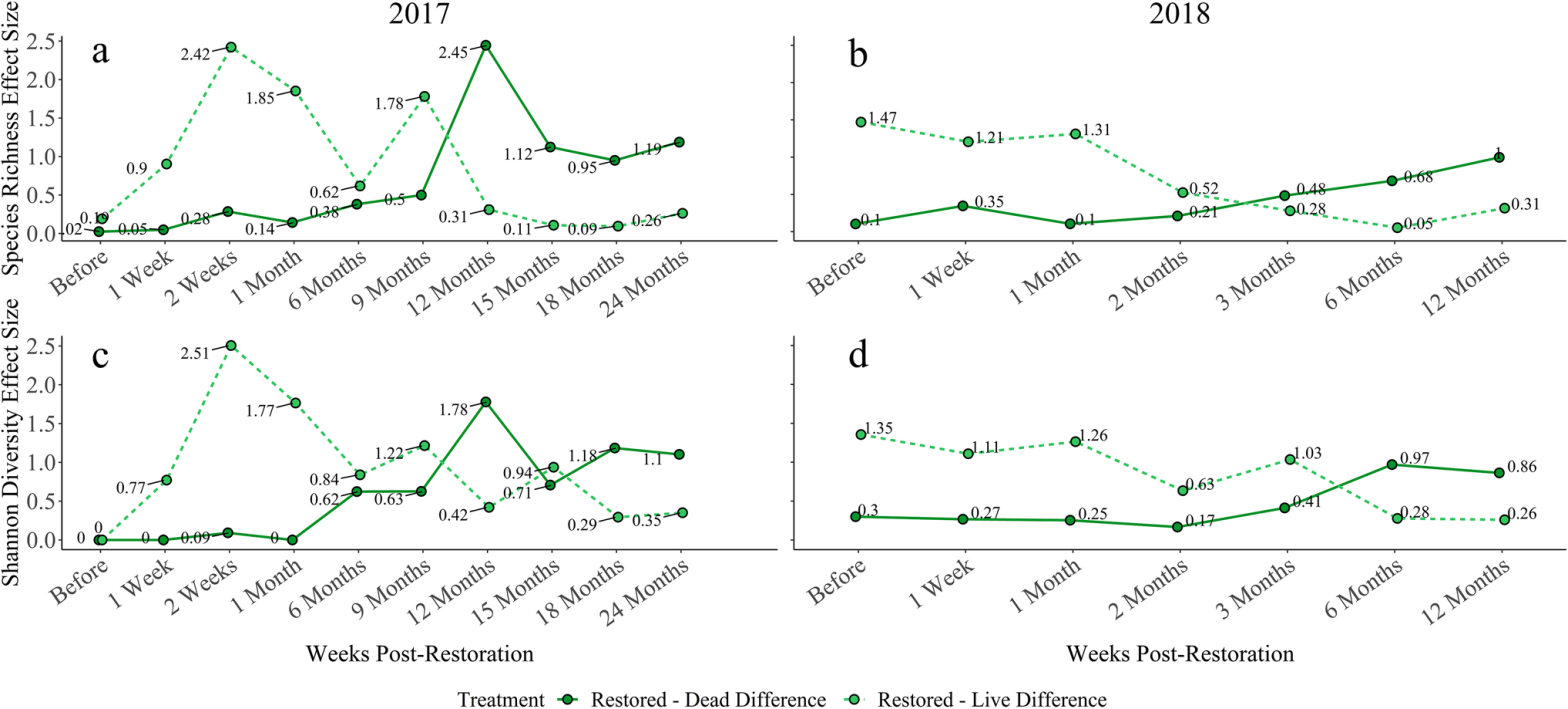
Effect sizes (Cohen’s D) of treatment on diversity metrics between restored and dead reefs as well as restored and live reefs over time. Species richness on 2017 reefs (**a**), Shannon diversity on 2017 reefs (**c**), species richness on 2018 reefs (**b**), and Shannon diversity on 2018 reefs (**d**).

Both species richness and Shannon diversity were high on live reefs throughout the study period, excluding the before-restoration time period where diversity was low across all treatments (Fig. 1). Species richness and Shannon diversity on 2017 restored reefs stayed relatively low and were similar to those of dead reefs up to six months post-restoration (Fig. 1). Prior to six months, the differences between restored and live reef richness and Shannon diversity were large (Fig. 2). Between six and nine months, restored reef diversity metrics began to increase and become more distinct from those of dead reefs (Figs. 1 and 2). Richness and Shannon diversity continued to increase and the effect of treatment on the differences in diversity between restored and dead reefs was greatest 12 months following restoration (richness *d* increased from 0.5 to 2.45; Shannon *d* increased from 0.63 to 1.78). The substantial increase in species richness and Shannon diversity on restored reefs during this time period resulted in restored reefs becoming more similar to live reefs (richness *d* decreased from 1.78 to 0.31; Shannon *d* decreased from 1.22 to 0.42). As species richness and Shannon diversity increased over the remainder of the study, differences between live and restored reef diversity were small to moderate, while differences between restored and dead reefs were relatively large (Fig. 2).

Diversity trends on 2018 oyster reefs were similar to those on 2017 reefs (Fig. 1). Species richness and Shannon diversity were higher on live reefs compared to dead reefs throughout the sampling period. Species richness and Shannon diversity on 2018 restored reefs slowly increased following restoration and mirrored dead reefs for approximately three months. By six months post-restoration, restored reef diversity were more similar to live reefs (Figs. 1 and 2). After six months, differences between restored and dead reefs continued to increase, revealing a positive trend in the recovery of species richness and diversity over time.

Species diversity was meaningfully associated with local environmental conditions. Water clarity, oyster density, oyster shell height, reef thickness, salinity, and dissolved oxygen were identified as predictors of species richness on 2017 oyster reefs (AIC = 156.37, df = 209, R^2^ = 0.271). Shannon diversity of 2017 oyster reefs was likewise predicted by oyster shell height, reef thickness, and dissolved oxygen levels (AIC = 437.68, df = 212, R^2^ = 0.258). Species diversity on 2018 oyster reefs was best predicted by dissolved oxygen, reef thickness, and oyster density (AIC = 175.4, df = 158, R^2^ = 0.172). Shannon diversity on 2018 reefs was predicted by oyster density and reef thickness (AIC = −268.35, df = 159, R^2^ = 0.163).

**Figure 3:**
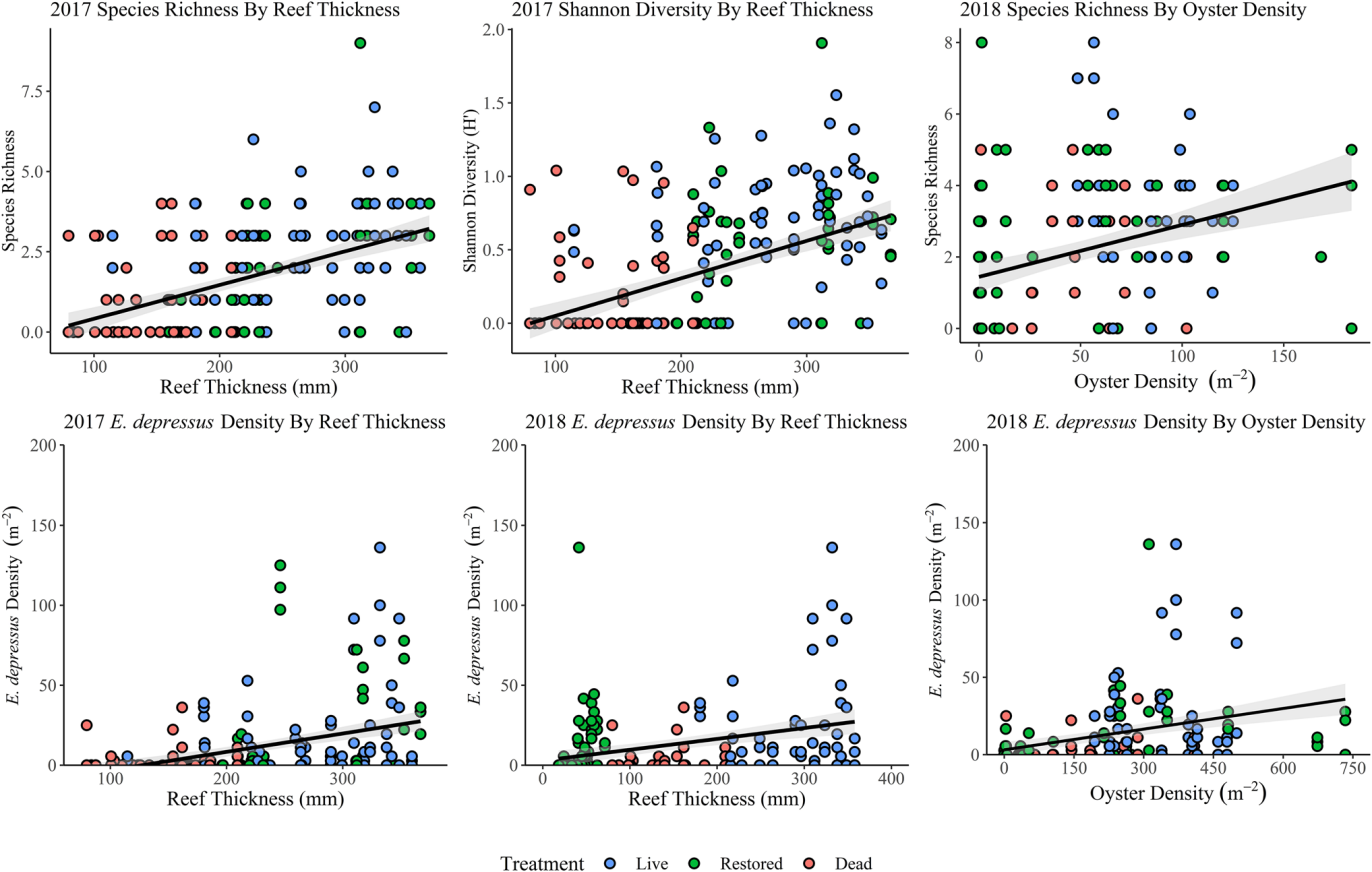
Scatterplots showing meaningful correlations between species diversity and abundance. Species diversity and abundance of *Eurypanopeus depressus* was significantly correlated with oyster reef thickness and oyster density. In general, these oyster habitat metrics increased from dead to restored to live reefs.

Direct correlations between species diversity and oyster reef habitats were also present. Firstly, species richness on 2017 oyster reefs was highly correlated with oyster reef thickness (r = 0.49, *p* < 0.001; Fig. 3) and weakly correlated with oyster reef density (r = 0.276, *p* < 0.001) and oyster shell height (r = 0.159, *p* = 0.019). The Shannon diversity of 2017 oyster reefs was also moderately correlated with reef thickness (r = 0.474, *p* < 0.001; Fig. 3) and oyster density (r = 0.248, *p* < 0.001). The species richness of 2018 reefs was also significantly correlated with reef thickness (r = 0.317, *p* < 0.001) and oyster density (r = 0.36, *p* < 0.001, Fig. 3). Both of these biotic variables were meaningfully correlated with Shannon diversity as well (r = 0.368, 0.331, *p* < 0.001, 0.001, respectively).

### Species composition

**Figure 4:**
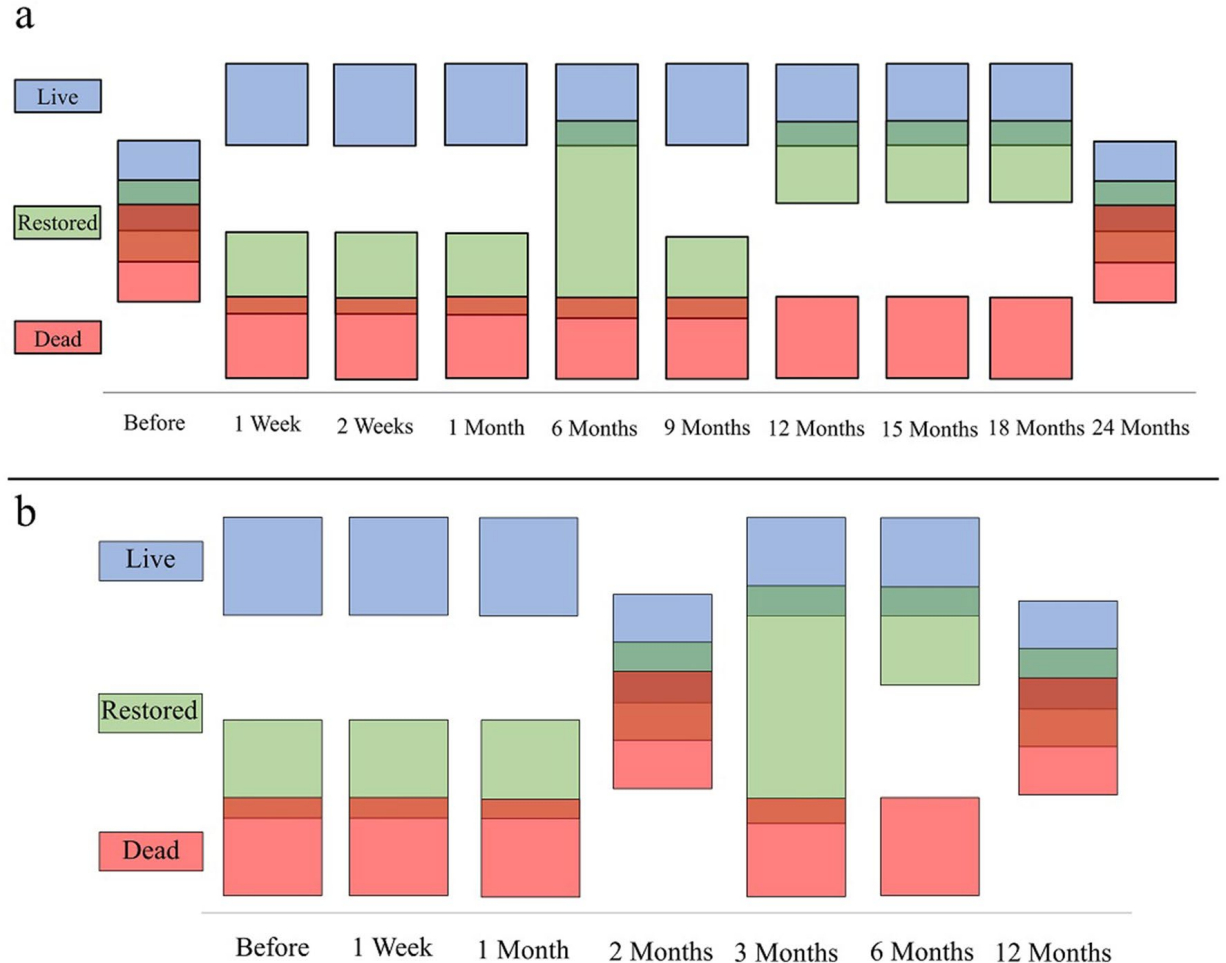
Illustrated results of PERMANOVA pairwise comparisons for 2017 reefs (**a**) and 2018 reefs (**b**). Overlapping squares represent no significant difference between live (blue), restored (green), or dead (red) reefs. Non-overlapping squares represent significant differences between treatments. Statistical significance is based on α = 0.05. Restored reef species composition generally began to reflect that of live reefs approximately 6 months after restoration. All squares overlap before restoration and 24 months after restoration on 2017 reefs and 12 months after restoration on 2018 restored reefs as there were no significant differences in species composition between treatments during these times. The lack of differences during these time periods are likely due to temporal variation in the populations of common crab species collected herein. Despite the lack of statistical differences during these time periods, recovery of species composition on restored reefs over the course of the study is still clear.

Noticeable shifts in species composition occurred on oyster reefs over time (Table 2). Species composition on 2017 reefs was significantly different between treatments and sampling times (PERMANOVA Pseudo-F_2,9_ = 42.716, 24.305, *p* = 0.001, 0.001). A significant interaction effect was also present (PERMANOVA Pseudo-F_18_ = 2.953, *p* = 0.001). Subsequent pairwise comparisons of treatments within sampling times revealed a shift in species composition on restored reefs from a dead-like state early in the restoration process to a live-like state over the course of the study (Fig. 4). Live reefs supported invertebrate communities that were statistically unique compared to dead reefs, save the before-restoration and 24-month sampling events wherein all treatments were similar. Species composition on restored reefs was not significantly different from dead reefs at 1 week, 2 weeks, and 1 month after restoration. During the 6-month sampling period the species composition of restored reefs was not significantly different than live or dead reefs. However, by 12-months post-restoration, the species composition of restored reef communities became significantly different from that of dead reefs and not significantly different than that of live reefs. This trend continued through to the 18-month sampling period (Fig. 4).

**Table 2:** Results of PERMANOVA pairwise comparisons of treatments within sampling times.

Groups	t	p (perm)	Permutations	Groups	t	p (perm)	Permutations
**2017 Oyster reefs**							
Before restoration				9 Months			
Live vs. dead	0.63	1	8	Live vs. dead	5.311	0.001*	762
Dead vs. restored	0.131	1	4	Dead vs. restored	1.522	0.208	16
Live vs. restored	0.615	1	11	Live vs. restored	2.887	0.006*	953
1 Week				12 Months			
Live vs. dead	2.982	0.002*	338	Live vs. dead	3.769	0.001*	990
Dead vs. restored	1.308	0.331	6	Dead vs. restored	3.998	0.001*	947
Live vs. restored	2.35	0.014	308	Live vs. restored	0.631	0.802	996
2 Weeks				15 Months			
Live vs. dead	3.205	0.003*	742	Live vs. dead	2.437	0.008*	994
Dead vs. restored	1.123	0.485	8	Dead vs. restored	2.715	0.01*	987
Live vs. restored	3.872	0.002*	390	Live vs. restored	1.501	0.107	996
1 Month				18 Months			
Live vs. dead	3.747	0.001*	691	Live vs. dead	2.649	0.005*	997
Dead vs. restored	0.848	0.633	18	Dead vs. restored	2.718	0.007*	985
Live vs. restored	2.958	0.003*	886	Live vs. restored	1.322	0.178	998
6 Months				24 Months			
Live vs. dead	2.525	0.012*	989	Live vs. dead	1.543	0.104	992
Dead vs. restored	1.17	0.243	907	Dead vs. restored	1.426	0.144	979
Live vs. restored	1.431	0.135	995	Live vs. restored	0.762	0.606	995
**2018 Oyster reefs**							
Before restoration				3 Months			
Live vs. dead	3.87	0.001*	989	Live vs. dead	2.423	0.01*	988
Dead vs. restored	0.722	0.612	126	Dead vs. restored	1.611	0.074	993
Live vs. restored	4.12	0.001	935	Live vs. restored	1.323	0.138	997
1 Week				6 Months			
Live vs. dead	2.152	0.019*	954	Live vs. dead	2.692	0.01*	997
Dead vs. restored	1.199	0.209	179	Dead vs. restored	1.958	0.033*	993
Live vs. restored	3.019	0.003*	837	Live vs. restored	1.256	0.188	999
1 Month				12 Months			
Live vs. dead	2.357	0.003*	996	Live vs. dead	1.543	0.104	994
Dead vs. restored	0.286	0.988	870	Dead vs. restored	1.771	0.066	998
Live vs. restored	2.291	0.012*	987	Live vs. restored	0.684	0.618	998
2 Months							
Live vs. dead	1.719	0.066	924				
Dead vs. restored	0.475	0.814	417				
Live vs. restored	1.585	0.08	937				

Restored reefs were statistically similar to dead reefs prior to 12 months in 2017. Species composition then shifted approximately 12 months after restoration and became statistically similar to live reefs. Species composition in 2018 shifted from a dead-like state to a live-like state within 6 months of restoration.

**Figure 5:**
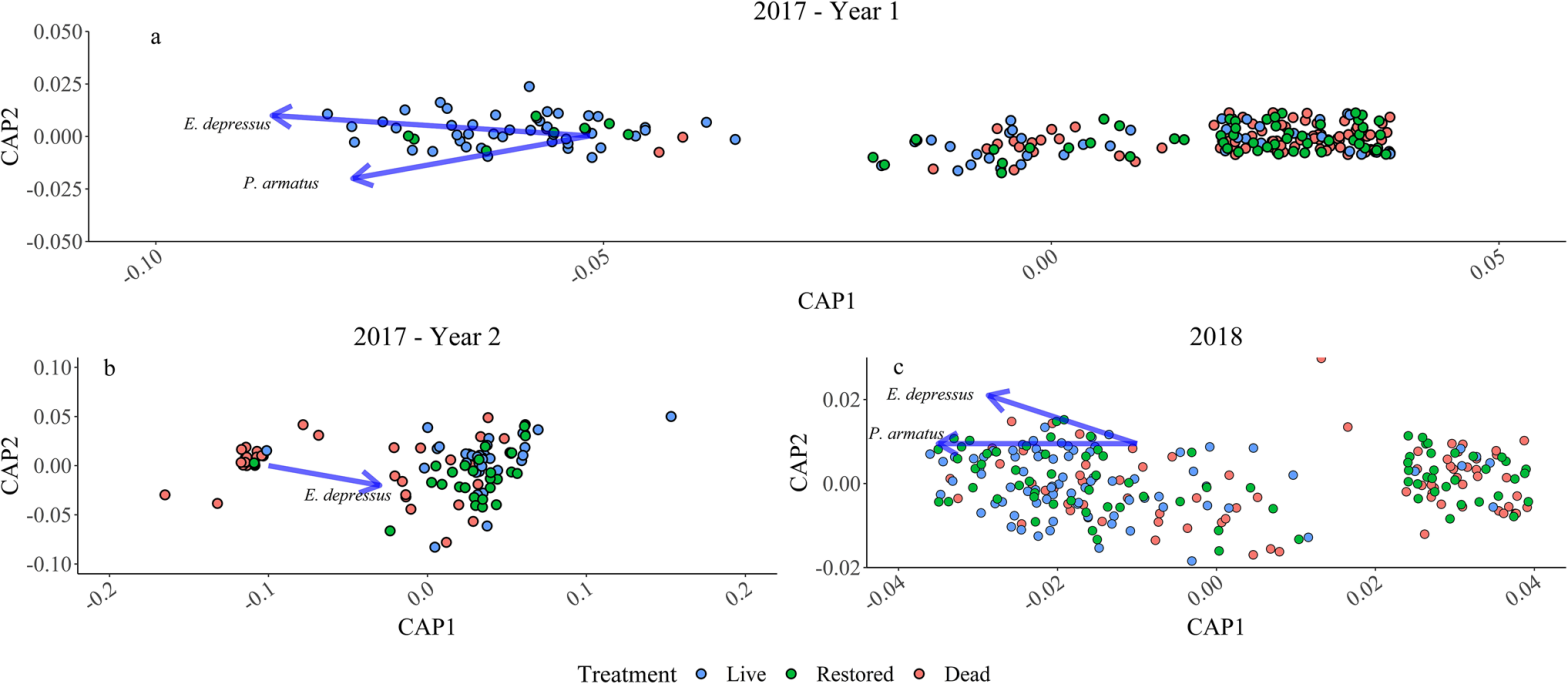
Canonical analysis of principle coordinated revealed meaningful effects of restoration on species composition. Overlayed vectors represent correlations of r = 0.8 or greater between CAP axes and species abundance. Eurypanopeus depressus abundance correlated with the effects of restoration in all ordiantions. Thus, E. depressus was selected as an indicator of restoration success.

Annual differences in species composition proved to be statistically significant for 2017 oyster reefs (i.e., Before—12-month vs. 15–24-month time periods; PERMANOVA Pseudo-F_1_ = 96.493, *p* = 0.001). Thus, to help isolate the restoration signal from potential statistical noise contributed by interannual variability, CAP was conducted separately for year 1 and year 2 data. Samples collected in year 1 displayed a moderate clustering pattern according to treatment along the CAP1 axis (Fig. 5). Samples collected on live reefs were generally separated from those collected on dead reefs, with restored reef samples spanning the two clusters. Overlayed species vectors suggested that *E. depressus* density was highly correlated with this axis while *P. armatus* density was moderately correlated with both axes. Similar results were observed in year 2 (Fig. 5). Samples were moderately to loosely separated by treatment along the CAP1 axis with high variability occurring across all samples along the CAP2 axis. The associated *E. depressus* vector was highly correlated with both axes.

Species composition on 2018 reefs was significantly different among treatments (PERMANOVA Pseudo-F_2_ = 19.912, *p* value = 0.001) and sampling times (PERMANOVA Pseudo-F_2_ = 12.046, *p* value = 0.001). There was also a significant interaction effect (PERMANOVA Pseudo-F = 1.643, *p* value = 0.031). Species composition on 2018 oyster reefs displayed a clear trend, moving from a dead-like state during early sampling to a live-like state at later time periods (Fig. 4). The species composition of restored reefs was not significantly different than that of dead reefs before, 1 week after, and 1 month after restoration. During the 2-month post-restoration sampling period, there were no significant differences among treatments. Restored reefs were simultaneously not significantly different from live or dead reefs during the 3-month sampling period. Species composition on restored reefs then shifted to become significantly different from dead reefs and not significantly different from live reefs at 6 months after restoration. Finally, all treatments were not statistically distinguishable from each other at 12 months post-restoration. Subsequent CAP revealed a slight clustering pattern among these samples (Fig. 5). *Eurypanopeus depressus* and *P. herbstii* vectors were correlated with both CAP1 and CAP2, while densities of *P. armatus* were highly correlated with CAP1 alone. However, high variation along both axes concealed any obvious trends in species composition.

Analysis of environmental parameters revealed several significant relationships between the physical environment and species assemblages. Species composition on 2017 oyster reefs was significantly correlated with ambient environmental conditions (RELATE; ρ = 0.095 *p* = 0.001). BEST analysis found oyster reef thickness alone yielded the best Spearman rank correlation between environmental parameters and species assemblages (ρ = 0.207). Additionally, the subsequent Distance Based Linear Model (DBLM) identified water temperature, salinity, water clarity, oyster reef thickness, and oyster shell height as significant predictors of species composition and accounted for 27.9% of the variation in the species assemblage (AIC = 1489, df = 214). The species composition of 2018 oyster reefs were significantly correlated with biotic and abiotic factors (RELATE; ρ = 0.086, *p* = 0.001). Subsequent BEST analysis revealed that oyster density alone yielded the greatest correlation with species composition patterns (ρ = 0.225). The associated DBLM identified water clarity, salinity, tidal height, oyster density, and reef thickness as meaningful predictors of species composition (AIC = 1133.8, df = 160, R^2^ = 0.267).

### Indicator species

**Figure 6:**
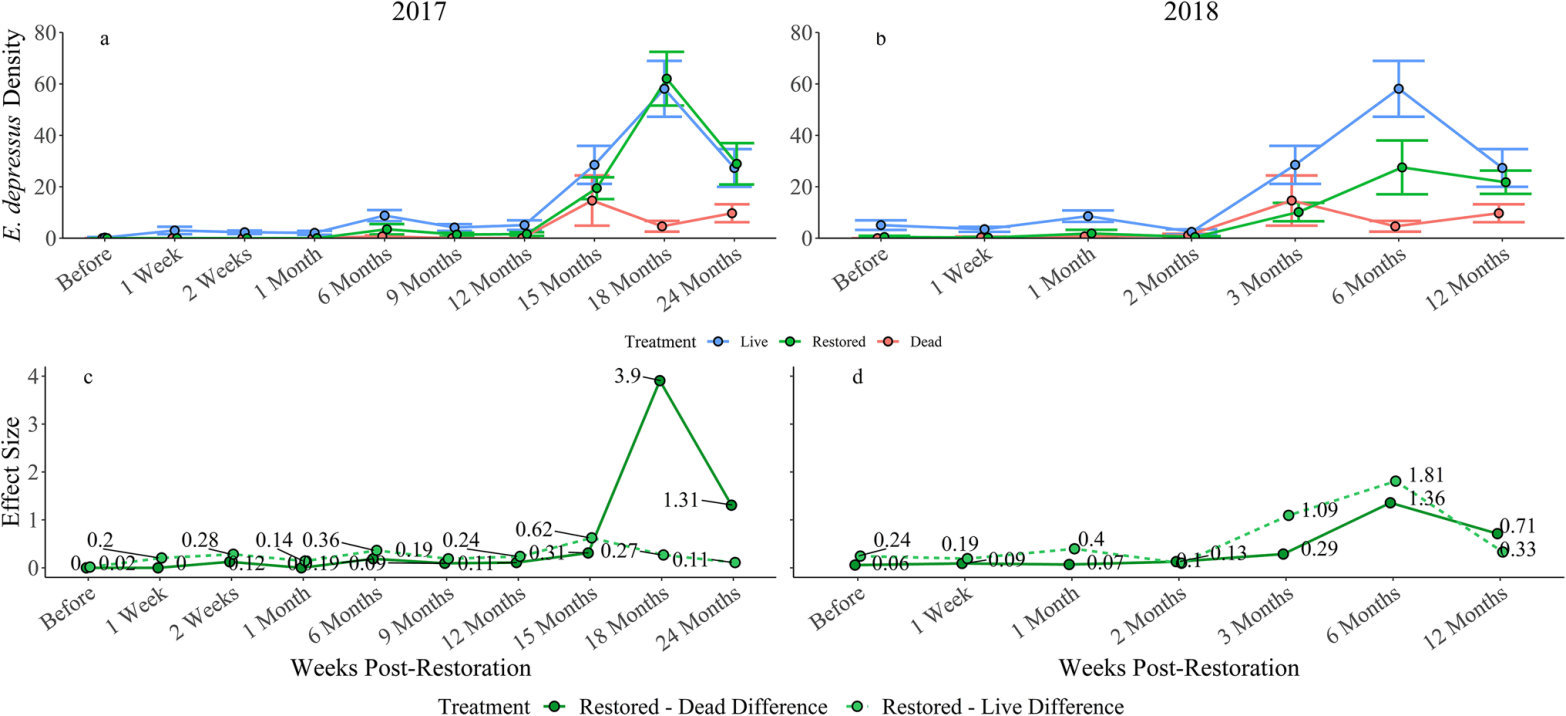
Density and effect size of treatment Eurypanopeus depresses over time. Abundance increased as soon as 6-months post-restoration. Abundance recovered fully after approximately 18 months after restoration. The effect of treatment on the difference between restored and dead reefs was low early in the study. However, the effect size increased over time. At the same time, the effect of treatment on the differences between restored and live reefs was small during this time.

*Eurypanopeus depressus* was selected as an indicator species based on consistent correlations with CAP axes associated with restoration effects (Fig. 5). *Petrolisthes armatus* was correlated with axes in all three ordinations but was not selected as a restoration indicator due to its status as a non-native species in ML^24^. The density of *E. depressus* on 2017 reefs was relatively low across treatments. However, *E. depressus* density was higher on live reefs compared to dead reefs for the entirety of the study (Fig. 6). The density of *E. depressus* was extremely low on dead reefs and restored reefs at 1 week, 2 weeks, and 1 month after restoration (d = 0, 0, 0.12; Fig. 6). However, density on restored reefs began to increase 6 months after restoration. The difference in *Eurypanopeus depressus* density between restored and dead reefs gradually increased from this time through the 15-month sampling period (d = 0.19–0.31). As mean density increased, restored reefs began to mirror live reefs 18 months post-restoration (d = 0.27). This trend continued through 24 months post-restoration (d = 0.11). The effect of treatment on differences in *E. depressus* densities on restored and dead reefs was quite large during these time periods (d ranged from 1.31 to 3.9).

Similar trends were observed on 2018 reefs (Fig. 6). Mean *E. depressus* density was higher on live reefs than dead reefs throughout the sampling period. The mean density of *E. depressus* on restored reefs mirrored that of dead reefs through the 3-month post-restoration sampling period (d ranged from 0.06 to 0.29; Fig. 6). Restored reef density then began to climb significantly 6 months after restoration and was comparable to that of live reefs during the 12-month sampling period (d = 0.33 SD). The effect of treatment on the differences between restored and dead reefs was quite large during this sampling period (d = 0.71 SD).

Indicator species density was associated with biotic and abiotic variables. Water clarity, reef thickness, salinity, water temperature, tidal height, and dissolved oxygen were identified as predictors of *E. depressus* density on 2017 oyster reefs (AIC = 1256.27, df = 209, R^2^ = 0.41, p < 0.001). Likewise, water clarity, tidal height, reef thickness, and water temperature were identified as meaningful predictors of *E. depressus* densities on 2018 reefs (AIC = 959.21, df = 157, R^2^ = 0.31, p < 0.001). Direct relationships between oyster reef habitat metrics and *E. depressus* densities were also detected. Specifically, 2017 *E. depressus* densities were moderately correlated with oyster reef thickness (r = 0.379, *p* < 0.001; Fig. 3) and 2018 densities were moderately correlated with oyster density and reef thickness (r = 0.359, 0.319, *p* < 0.001, 0.001; Fig. 3). Further exploration revealed trends in *E. depressus* densities across oyster reef treatments. In general, *E. depressus* densities increased as reef thickness and oyster densities increased, all of which tended to be highest on live reefs and lowest on dead reefs.

## Discussion

Restoration of intertidal oyster reefs resulted in an increase in oyster density and macroinvertebrate species abundance, community diversity, and composition. Reef macroinvertebrate communities responded overwhelmingly positively to habitat restoration. Community metrics on restored reefs mirrored those of live reefs, in some cases, as soon as one-year post-restoration. Species diversity and abundance were also significantly positively correlated with oyster reef habitat metrics, such as reef thickness and oyster density, which were higher on live and restored reefs than on dead reefs. Furthermore, several oyster reef habitat variables, in addition to other abiotic predictors, explained significant amounts of variation in species composition across treatments.

Restoration appears to have positive effects on macroinvertebrate communities inhabiting intertidal oyster reef interiors. Species richness and Shannon diversity on restored reefs increased and began to mirror trends on live reefs within one year of restoration. Species richness and Shannon diversity on 2017 restored reefs in particular were nearly identical to respective live reef diversity values after the 12-month sampling time. Species composition on restored reefs likewise became similar to live reefs within one year of restoration. These results suggest oyster reef restoration can facilitate the recovery of species diversity and composition. Similar studies conducted in other estuaries have likewise documented increases in densities of resident invertebrates and fishes post-restoration^29,31–33^. For example, Jud and Layman^29^ report the convergence of resident fauna biomass on a restored oyster reef and natural reef controls in the Loxahatchee River Estuary within 22 months after restoration. Conversely, studies in other estuaries including Caillou Lake, Louisiana and Mobile Bay, Alabama did not observe sustained growth in invertebrate densities within the temporal confines of their experiments (i.e., within 21–36 months). These discrepancies suggest that the timing and trajectory of functional recovery likely varies among systems and landscape settings^33,34^. Therefore future research efforts should aim to accurately quantify response and recovery times following restoration, to facilitate the development of more effective and targeted restoration and monitoring plans.

Differences in post-restoration macroinvertebrate community dynamics on reef margins and interiors are most probably a result of environmental processes. Reef margins represent transition zones between the complex three-dimensional habitat created by oysters and surrounding bare bottom habitats. Edge effects, and associated statistical noise in time series data, may have prevented the detection of clear restoration trends on reef margins. These edge effects, such as diminished oyster densities and prolonged tidal submergence, may influence macroinvertebrate assembly uniquely on reef margins^35,36^ and thus lead to community-level differences between these two oyster reef zones. The effect of these environmental differences on oyster density have been documented^36^. However, the impact of these edge effects on fine-scale macroinvertebrate community zonation has been given little attention. Given data in–hand, an in-depth investigation of this phenomenon is beyond the scope of this study, but warrants further investigation. An exploration of fine-scale zonation of species diversity and composition between intertidal oyster reef interiors and margins in future studies would provide insight into this poorly understood phenomenon and may have major implications for future monitoring programs, restoration efforts, and experimental designs targeting invertebrates inhabiting oyster reefs.

Given the observed post-restoration trends, the case for the positive effects of restoration is strong. Yet, it is possible that recovery trends may have been partially influenced by the overall increase in species diversity observed towards the end of the study. Elevated diversity levels observed 15–24 months after restoration were likely a result of natural variation as they were observed across all treatments. Seasonal and interannual cycles in the abundance and diversity of estuarine invertebrates have been previously recorded in these communities^37,38^ and elsewhere in the world^39–41^. However, the temporal scale of this study may not have allowed for the quantification of such patterns. Longer-term temporal variability in invertebrate communities may have thus influenced the results of the last three sampling events in our, relatively speaking, short-term dataset. This would explain elevated diversity on dead reefs 15–24 months after restoration as well as the similarity in species composition during the 24-month sampling time. Despite the natural variation in invertebrate diversity affecting all treatments, species composition still trended towards a live reef-like state over time. Moreover, the effect of restoration on differences between restored reefs and dead reefs was still strong during these times, while the differences between restored and live reefs remained low. Thus, restoration was likely the primary driver of recoveries in community-level metrics, independent of the variability characteristic of these communities.

Restoration of dead oyster reefs also had a noticeable effect on species-specific abundance. Restoration trends were apparent, though seasonal variability was present. *Eurypanopeus depressus* abundance tended to peak in winter months (6- and 18-months post-restoration). Abundance maximums during winter were a surprising finding. However, water temperature may not be as important in determining the survival and growth of *E. depressus* as other seasonal abiotic conditions. According to the findings of Van Horn and Tolley^42^, *E. depressus* distribution in another Florida estuary is influenced by salinity regimes as the panopeid was most abundant in moderate salinity environments^42^. Furthermore, temperature has been shown to have only minor effects on the survival, growth, and dispersal of oyster reef-dwelling mud crab larvae while salinity tends to exert consequential influence on these life history processes^43,44^. Salinity is relatively low in ML during winter months (~ 25 to 30 ppt) compared to summer (~ 35 to 40 ppt)^45^. The moderate salinities characteristic of ML winters may thus be beneficial for *E. depressus* populations, despite low temperatures. The anomalous spike observed 18 months after restoration (6 months after for 2018 reefs), was likely a result of favorable environmental conditions interacting with the aforementioned seasonal and interannual variation in invertebrate diversity and abundance^37,38^. Despite this variability, the recovery of *E. depressus* abundance on restored reefs was clear. Positive correlations with reef thickness and oyster density further suggest that the effect of restoration was meaningful. As oyster reef complexity increased so too did habitat suitability for *E. depressus*. Jud and Layman^29^ experimentally tested the effects of habitat complexity on benthic oyster reef communities by enumerating organisms present on high relief and low relief oyster tray treatments in a nearby Florida estuary. High relief habitats tended to support higher biomass, diversity, and abundance compared to low relief habitats, corroborating our in situ correlations^29^. Thus, the effects of habitat complexity on *E. depressus* abundance are likely meaningful, further suggesting the development of oyster reefs following restoration results in the recovery of *E. depressus* populations.

*Eurypanopeus depressus*, in addition to species diversity, likely serves as an excellent indicator of restoration success due to its clear recovery trend over time, close association with increasing oyster habitat complexity, and correlation with restoration effects in multivariate ordination space. But why was *E. depressus* the only native species that consistently responded to restoration? We believe the answer lies in the natural histories of these oyster reef invertebrates. Of the 41 species enumerated herein, three of them are oyster reef-obligates^24,46^. The other 38 are generalists in their habitat requirements and can also be found inhabiting other coastal habitats such as bare-bottoms, seagrasses, mangroves, and salt marshes^47–52^. There is no doubt the restoration of oyster reefs is beneficial to the reef-obligate species as it provides high quality habitat. However, *E. depressus*, *P. herbstii*, and *Panopeus simpsoni* (oystershell mud crab Rathbun 1930) are directly reliant on oyster reef and shell-based habitats as their sole means of foraging grounds, reproductive opportunities, and refugia from predation^24,25,46,53^. Structure-reliant, habitat-specific species have been shown to recover relatively rapidly compared to more generalist and mobile species in this region^54^. Harris^54^ found infaunal invertebrates responded almost immediately to restoration in ML and the abundance of several taxonomic groups increased drastically within one month of restoration. Conversely, Shaffer et al.^55^ found the abundance of wading birds, highly mobile predators, was similar among reef types in ML when conducting space-for-time observations. Thus, we would expect reef-reliant, low mobility species to respond rapidly to habitat restoration, as *E. depressus* did. However, *P. herbstii* and *P. simpsoni* were not correlated with CAP axes associated with restoration effects. The reason for this discrepancy is unclear but seasonal or interannual variation may have acted to cloud any relationships between these species and the effects of restoration. Furthermore, the generation time of *P. herbstii* is approximately 1 year, which is twice that of *E. depressus*^25^. *Panopeus herbstii* also produces fewer broods per generation. The decreased lifetime and greater reproductive output of *E. depressus* likely enables it to more effectively colonize to new habitats via larval dispersal. The importance of larval dispersal to restoration success has been documented several times for sessile species, including *C. virginica*^56–58^. However, the relationship between larval dispersal and recovery of mud crab populations post-restoration, to our knowledge, has never been assessed. If given more time *P. herbstii* and *P. simpsoni* may colonize restored reefs and their relationship with restoration effects may be more easily detected. Despite this disconnect, *E. depressus* abundance served as an excellent measure of restoration success. Thus, habitat-obligate species appear to be reliable and positively correlated indicators of restoration success. However, other factors such as interannual variation, life history parameters, and reef-scale species distributions likely regulate the direct response of these species to habitat restoration. The findings of this study suggest research and monitoring programs employing these species as indicators of restoration success sample the interiors of nearby dead and live reefs to enable relative comparisons, and more accurately capture differences among treatments (i.e., samples from reef interiors generated less noise regarding differences than samples collected from reef margins). By comparing indicator species abundance between restored and reference reefs, scientists can effectively track restoration success while accounting for natural variation introduced via interannual and seasonal population dynamics.

Operationalizing the concept of single species as indicators of restoration success requires relatively robust metrics and methods to identify suitable indicator species a priori. In this study, the numerical response of *E. depressus* to oyster reef restoration serves as an excellent case study on the utility of habitat-obligate species in post-restoration assessments. The analyses outlined and conducted herein to identify *E. depressus* as an effective indicator were relatively complex. However, this methodology provides a conceptual framework grounded in the natural history and ecology of *E. depressus* that may be translated to other species and systems. To identify species that may serve as responsive indicators of restoration success we propose identifying species that are: (1) native to the system in which restoration efforts are taking place, (2) habitat-obligates or species requiring relatively high quality habitat of the type being restored, (3) highly abundant relative to other native habitat-dependent species, (4) relatively abundant year-round (i.e. to minimize potential influences of episodic settlement pulses rapidly reduced through post-settlement mortality), (5) tolerant of seasonal fluctuations in influential abiotic regimes (e.g., salinity and temperature), and (6) easily identified and enumerated in the field. These characteristics represent criteria against which natural resource managers may screen local species inventories to identify species that could serve as effective indicators of restoration success. Managers may then infer the status of habitat restoration success over time by monitoring the abundance of these species. The implementation of this framework in management strategies and monitoring programs will provide simple, easily collected, and comparable metrics for managers to efficiently assess the success of restoration efforts across ecosystems. Thus, this highly transferable framework will augment the efficiency of post-restoration monitoring efforts, thereby improving our ability to understand the recovery of ecological communities and broader ecosystem function in restored habitats.

The recovery of macroinvertebrate communities benefits ecosystem function beyond oyster reef habitats. Wong et al.^23^ found oyster reefs accounted for the greatest secondary production among all estuarine habitat types. Annelids, arthropods, and molluscs were responsible for the vast majority of this production. In addition to contributing to the immense secondary production of oyster reefs, the macroinvertebrates studied herein efficiently transfer this production to high trophic levels. Specifically, *E. depressus* feeds on both microphytobenthos as well as small crustaceans and bivalves, while *P. herbstii* feeds solely on bivalves and small crustaceans^24,25^. Bivalves directly assimilate pelagic production via filter feeding^59,60^. These trophodynamic species thus serve as direct or near-direct links to both benthic and pelagic production. Higher level predators that prey on these species, including *C. sapidus*, snappers, drum, and predatory wading birds, benefit from efficient energy transfer through minimal trophic levels^55,59–64^. These species thus contribute significantly to the biomass pool of high trophic levels, including ecologically and commercially important fisheries^60,64^. Oyster reef restoration thus has the potential to significantly augment fisheries production as well as ecological function of estuarine systems. Estimates of the relative importance of these mid-trophic level organisms to broader estuarine ecosystem structure are lacking. However, given the high secondary production and trophic efficiency these species provide to estuarine food webs it is reasonable to assume their contributions are ecologically important.

In conclusion, oyster reef restoration has positive impacts on resident macroinvertebrate communities. This study provides an in-depth view into the post-restoration dynamics of these communities via comparison to both natural and dead reef states. Additionally, our analyses identify species diversity and habitat-specific species abundance as effective indicators of restoration. This study yields restoration timelines of 1 year for the recovery of species diversity and composition and at least 18 months for species abundance in this subtropical estuary. These response times are likely dependent on seasonal and interannual environmental variation. Nevertheless, successful oyster reef restoration facilitates the recovery of ecologically crucial macroinvertebrates. These organisms fill ecologically crucial roles by shortening food chains and efficiently transferring energy from basal resources to top-level predators, including economically important fishes and predatory birds. Understanding their response to restoration efforts is thus vital to broader ecosystem-based management of some of the most productive and anthropogenically impacted ecosystems in the world, our estuaries.

## Methods

### Study area

Mosquito Lagoon (ML; 28.835940˚ N, 80.796794˚ W) is the northernmost basin of the Indian River Lagoon (IRL) system and is connected to its remainder via Haulover Canal, a man-made channel located in the southern half of ML. Ponce De Leon Inlet defines the northern end of ML and is the basin’s only ocean access. Mosquito Lagoon supports a complex habitat matrix that includes 2542 intertidal oyster reefs composing 46.34 ha^65^. Furthermore, due to its location in a biogeographic transition zone, the IRL is one of the most diverse estuaries in North America and is home to over 400 fish species (of the 782 species found along the entirety of the east coast of central Florida) as well as several hundred species of invertebrates, birds, and marine mammals^66–70^.

### Oyster reef restoration

The methods replicated in this study for oyster reef restoration have been previously successful in ML^65^. Dead reef sites, which are composed of disarticulated oyster shell stacked over a meter above the water’s surface, assigned to the restoration treatment were leveled to the intertidal zone by four to six-person teams using shovels and pickaxes. Disarticulated oyster shells were attached to aquaculture-grade Vexar™ mesh mats (36 shells per 0.25 m^2^ of mat) and placed on the leveled reef area. Each corner of the oyster mats was then anchored to donut weights and zip-tied together in a quilt-like fashion. This procedure anchored the developing reef, making it resistant to uprooting and toppling by natural wave action and anthropogenic boat wakes^65^. Restored reefs were then able to develop into living oyster reefs that support equal densities of oysters as their natural counterparts^65^. Unfortunately, historic oyster density does not exist for ML. Oyster reefs from this region appear similar in areal imagery from 1943 and 2009^65^. However, modern reefs are likely degraded compared to historical conditions due to anthropogenic pressures such as eutrophication and exploitation^6,71^. Nevertheless, restored reefs in ML have consistently achieved restoration targets of 1000 oysters/m^2^, which is considered equivalent to natural counterparts^72^. These restoration methods and criteria were applied to four dead oyster reefs in May 2017 and an additional 4 dead reefs in May 2018.

### Sampling design

A Before-After-Control-Impact (BACI) design was implemented on a total of 16 oyster reefs starting in May 2017. In the summer of 2017, four dead reefs were designated for restoration, four dead reefs were designated as negative controls, and four live reefs were selected as positive controls. Live control reefs consisted of four patch reefs, dead controls consisted of four patch reefs, and all reefs designated for restoration in 2017 were patch reefs, while those restored in 2018 were fringing reefs associated with mangrove shorelines. Reef type can have significant impacts on species diversity and composition^73^. However, reef type was not included in final model runs as the variation in diversity indices attributable to site-level effects regularly approached zero. Each site was sampled over the course of two years at the following time periods: one week pre-restoration as well as one week, two weeks, one month, six months, nine months, 12 months, 15 months, 18 months, and 24 months post-restoration. Planned two-month and three-month sampling events were lost due to damages caused by Hurricane Irma. The same design was employed for a second set of oyster reefs restored in the summer of 2018. Reefs used as positive and negative controls for the reefs restored in 2017 were again used for comparison to the reefs restored in 2018. Reefs sampled in conjunction with the 2018 restoration effort were sampled one week before restoration as well as one week, one month, three months, six months and 12 months after restoration. The reefs restored in 2017 and 2018 are referred to as 2017 reefs and 2018 reefs, respectively.

Oyster reefs were sampled for macroinvertebrates using lift nets, which were originally designed by Crabtree and Dean^74^ and modified for use in Florida by Tolley & Volety^18^. They were constructed using PVC and 2 mm mesh netting. The PVC was assembled into 0.6 m × 0.6 m square frames and a 0.5 m deep mesh net (2 mm mesh size) was attached using Zip Ties. Six nets were deployed on each oyster reef. Three lift nets were placed on each oyster reef interior (~ 1 m away from edges, near the mean high tide line) and three on each reef margin (adjacent to reefs within ~ 0.5 m, near the mean low tide line) to ensure thorough sampling of these complex habitats. Intertidal oyster reef interior and margin samples were collected simultaneously, processed following the same methods, and analyzed with standardized statistical techniques. Each net held an oyster reef mat identical to those use in the restoration process. These mats simulated habitat cover for fish and invertebrates and have been found to be highly effective at sampling oyster reef invertebrate populations^37^. Lift nets were deployed one week prior to sampling. Upon collection, researchers retrieved nets by slowly approaching them on foot and pulling up swiftly to trap any fauna utilizing the disarticulated oyster shells. Their accompanying oyster mats were left in place in the water near the study sites. All invertebrate species captured that were able to reach a max size of > 5 mm were stored in 70% ethanol and brought back to the lab for identification and enumeration. Simultaneously, salinity, water temperature, water clarity, and dissolved oxygen were measured at each site at each sampling period using a handheld multiparameter probe. Oyster density (m^−2^), oyster shell height (mm), and reef thickness (= canopy height; mm) were likewise recorded as biotic environmental variables at each sampling time according to the methods outlined in Baggett et al.^75^ and Chambers et al.^76^. At each sampling event, five 0.25 quadrats were thrown haphazardly on each reef. The number of live oysters within these quadrats was averaged and used to calculate density at each reef. The shell height of fifty oysters from each of these quadrats was also measured and averaged to obtain an average shell height for each reef. In addition to these quadrats, an additional replicate was placed on the highest point of each quadrat. Reef thickness was then measured for each quadrat and the mean of these values was calculated to arrive at the average reef thickness.

### Data analyses

During field sampling one of the oyster reefs restored in 2017 was lost to physical disturbance (i.e., hurricane), which resulted in erosion and the reef returning to a dead state. This reef was thus excluded from analyses. Species richness and Shannon–Wiener diversity were calculated based on collected abundance data. Shannon diversity was used in addition to species richness to ensure species evenness was considered^77,78^. Due to the high number of zeroes in our data, estimation of other evenness-based diversity metrics, such as Simpson’s diversity and Pielou’s evenness were not appropriate. Linear mixed effects models from the nlme package in R^79^ were used to test the effects of treatment and sampling time on each measure of diversity and indicator species abundance. Individual reefs were included as a random variable to provide sufficient power to detect differences among treatments and to account for potential spatial pseudo-replication^80–82^. Multiple pairwise comparisons were used to calculate estimated marginal means (emmeans package in R)^83^ and, Cohen’s d was used to measure effect size for differences in diversity responses between treatments within sampling periods. The resultant effect sizes were used to infer the effect of restoration on species diversity.

To ensure a comprehensive analysis of the effects of restoration, changes in species composition post-restoration were also quantified. Prior to analysis, all data were fourth root transformed to reduce the influence of dominant species. Bray–Curtis similarity matrices were then calculated using a dummy variable of 1 to stabilize dispersions in the data^84^. Permutational multivariate analysis of variance (PERMANOVA) was used to formally test hypotheses regarding changes in species composition post-restoration. PERMANOVA tests hypotheses via permutations of Bray–Curtis similarity matrices^85^. Canonical analysis of principal coordinates (CAP) was performed following PERMANOVA results. CAP is a discriminate ordination method used to graphically display statistically validated differences between groups based on Bray–Curtis similarity matrices^85–87^. Vectors representing correlations between species’ abundances and ordination axes were overlayed on resultant CAP ordinations to identify species indicative of restoration effects. A cutoff correlation of r = 0.75 was used to select species highly correlated with the effects of restoration. Linear mixed effects models were subsequently used to detect differences in indicator species abundance among treatments and time. The combination of PERMANOVA and CAP enabled the quantification of community-level responses to restoration as well as the identification of species driving these responses.

A suite of analyses was used to relate species composition, diversity, and indicator species abundance responses to corresponding environmental metrics. The following environmental parameters were included in analysis: water clarity (m), salinity (ppt), water temperature (℃), tidal height (m), reef thickness (mm), oyster density (m^−2^), and oyster shell height (mm). The Primer-e RELATE function was first used to detect the existence and strength of any relationship between environmental variables and species assemblages. The RELATE function first compares divergences of assemblages and environmental variables across samples. Subsequently, this function quantifies the strength of the relationship between them, yielding a Pearson rank correlation (ρ)^87^. However, the RELATE function lacks the ability to identify specific variables contributing to this rank correlation. Thus, upon a significant RELATE results the BEST function (Primer-e) was used to identify the combination of variables that yielded the strongest Pearson rank correlation. The BEST function utilizes a stepwise search of environmental parameters to maximize the rank correlation between environmental and biotic similarity matrices. Finally, distance-based linear models (DBLM) were constructed to quantify the amount of variation in species assemblages explained by corresponding environmental parameters (R^2^)^87,88^. Multiple linear regression was likewise used to quantify the effects of abiotic variables on diversity and indicator species abundance. Backwards stepwise selection based on Akaike information criterion (AIC)^88^ was used to identify distance-based and linear models that explained the greatest amount of information while preserving model parsimony^89^. Furthermore, simple Pearson correlation coefficients were calculated to evaluate direct relationships between restoration-based metrics (i.e., reef thickness, oyster density, and oyster shell height) and species diversity and abundance. These combined analytical techniques facilitated the development of a comprehensive view of community-diversity- and species-level responses to oyster reef restoration.

### Ethics declarations

Ethical approval or informed consent are not applicable to low-level invertebrates.

## Data Availability

The datasets used in this investigation are available from the corresponding author upon reasonable request.
